# Prognostic value of long non-coding RNA breast cancer anti-estrogen resistance 4 in human cancers: A meta-analysis

**DOI:** 10.1097/MD.0000000000015793

**Published:** 2019-05-24

**Authors:** Yang Meng, Yu-Lan Liu, Kai Li, Tao Fu

**Affiliations:** a Department of Gastrointestinal Surgery II, Key Laboratory of Hubei Province for Digestive System Disease; b Department of Critical Care Medicine, Remin Hospital of Wuhan University, Wuhan, China.

**Keywords:** long non-coding RNA breast cancer anti-estrogen resistance 4, meta-analysis, neoplasm, prognosis

## Abstract

**Background:**

Since long non-coding RNA breast cancer anti-estrogen resistance 4 (lncRNA BCAR4) is dysregulated in various types of cancers, we conducted a meta-analysis to determine its prognostic value in cancer.

**Methods:**

PubMed, EMBASE database, and CENTRAL were systematically searched.

Pooled hazard ratios (HRs) with 95% confidence intervals (CIs) were collected to estimate the prognostic value. Odds ratios (ORs) and their 95% CIs were used to assess the association between lncRNA BCAR4 expression and clinicopathological features, including tumor size, differentiation, lymph node metastasis, distant metastasis, and tumor stage.

**Results:**

Ten studies with 890 patients were included in this meta-analysis. The pooled results indicated that high lncRNA BCAR4 expression was associated with poor overall survival (OS) (HR 2.80, 95% CI: 2.08–3.78; *P* < .001). Overexpression of lncRNA BCAR4 was related to lymph node metastasis (OR 3.68, 95% CI: 2.25–6.00; *P* < .001), high tumor stage (OR 3.19, 95% CI: 1.98–5.13; *P* < .001), and distant metastasis (OR 3.83, 95% CI: 2.15–6.82; *P* < .001), but not to tumor size.

**Conclusions:**

Therefore, lncRNA BCAR4 overexpression is associated with poor OS and advanced clinicopathological features, and lncRNA BCAR4 may be a novel prognostic biomarker in cancer patients. However, further high-quality studies are needed to confirm these findings.

## 1 Introduction

In recent years, non-coding RNAs have been shown to play a significant role in the organization and regulation of genome.^[1–3]^ Non-coding RNAs are classified in 2 main groups: small non-coding RNAs (miRNAs) and long non-coding RNAs (lncRNAs, >200 nucleotides in length). Many lncRNAs, such as HOTAIR, UCA1, and MALAT1 have been demonstrated as significant regulator of tumor development and progression.^[4–7]^ LncRNA BCAR4 (breast cancer anti-estrogen resistance 4) was reported to be related to hormone resistance in breast cancer.^[8]^ Current researches have found that lncRNA BCAR4 promotes breast cancer proliferation and metastasis by regulating Hedgehog/GLI2 pathway, and contributes to anti-estrogen resistance.^[9,10]^ LncRNA BCAR4 was highly expressed in several tumors, including breast cancer, colon cancer, osteosarcoma, and non-small cell lung cancer.^[11–15]^ Moreover, the expression of lncRNA BCAR4 was suggested to be associated with various tumor biological parameters, including metastasis and prognosis.^[13,16]^ Ju et al^[17]^ found that high lncRNA BCAR4 expression was associated with distant metastasis and overall survival but not with tumor size in osteosarcoma. Gong et al^[18]^ indicated that lncRNA BCAR4 upregulation in non-small cell lung cancer was related to TNM stage, lymph node metastasis, and overall survival but not correlated with tumor size and histological grade. The prognostic value of lncRNA BCAR4 in cancer patients has varied among studies. Therefore, we conducted this meta-analysis to explore the relationship between lncRNA BCAR4 overexpression and prognosis in cancer.

## 2 Materials and methods

### 2.1 Search strategy

This meta-analysis was performed in accordance with the Preferred Reporting Items for Systematic Reviews and Meta-Analyses guidelines.^[19]^ As this study was a review of previous published studies, ethical approval or patient consent was not a requirement. An electronic search of PubMed, Embase, and CENTRAL for all relevant studies was conducted, with the last search ran on January 10, 2019. The key words used for the searches were as follows: “BCAR4” or “breast cancer anti-estrogen resistance 4” or “lncRNA BCAR4” and “cancer” or “neoplasm” or “tumor.” Only studies published in English were included. References from retrieved articles were also examined for additional relevant studies.

### 2.2 Selection criteria

Studies that met the following criteria were included: the relationship between lncRNA BCAR4 expression and clinicopathological features and prognosis was reported; lncRNA BCAR4 expression was measured in human cancer tissues, and patients were grouped according to lncRNA BCAR4 expression; the hazard ratios (HRs) and 95% confidence interval (CI), raw data, or survival curves were provided. Case reports, letters, review articles, conference abstracts, and laboratory studies were excluded. For studies that reported results of the same or overlapping data, only the study with the largest sample size was included.

### 2.3 Data extraction and quality assessment

Two reviewers extracted data from eligible studies independently. The following information was extracted: the name of the first author, the year of publication, country, tumor type, sample size, clinicopathological features, and outcomes of statistical analyses. The quality of all included studies were assessed according to the Newcastle-Ottawa scale (NOS score).^[20]^ Articles with a NOS score ≥6 were deemed of high quality. Any disagreement was resolved by discussion.

### 2.4 Statistical analyses

The HR was used as a measure of the association between lncRNA BCAR4 expression and cancer patients prognosis. We used the HRs and 95% CIs reported in the original article when it was available and when they were not reported but with Kaplan–Meier curve, the HR values were extracted from a Kaplan–Meier curve by using Engauge Digitizer (V.4.1).^[21]^ Odds ratios (ORs) and their 95% CIs were used to assess the association between lncRNA BCAR4 expression and clinicopathological features, including tumor size, differentiation, lymph node metastasis, distant metastasis, and tumor stage. Between-study heterogeneity was measured by the *Q* and *I*^2^ tests. A probability value of *I*^2^ >50% or *P* < .1 indicated the existence of significant heterogeneity.^[22]^ A random effects model or fixed effects model was selected based on the results of heterogeneity analysis. The random-effects model would be used if heterogeneity was significant, otherwise the fixed effects model would be used. The potential publication bias was assessed by the Begg funnel plot. Subgroup analyses were conducted to explore potential sources of heterogeneity. All statistical analyses were performed with RevMan 5.3 software (The Cochrane Collaboration, Oxford, UK).

## 3 Results

### 3.1 Characteristics of eligible studies

**Figure 1:**
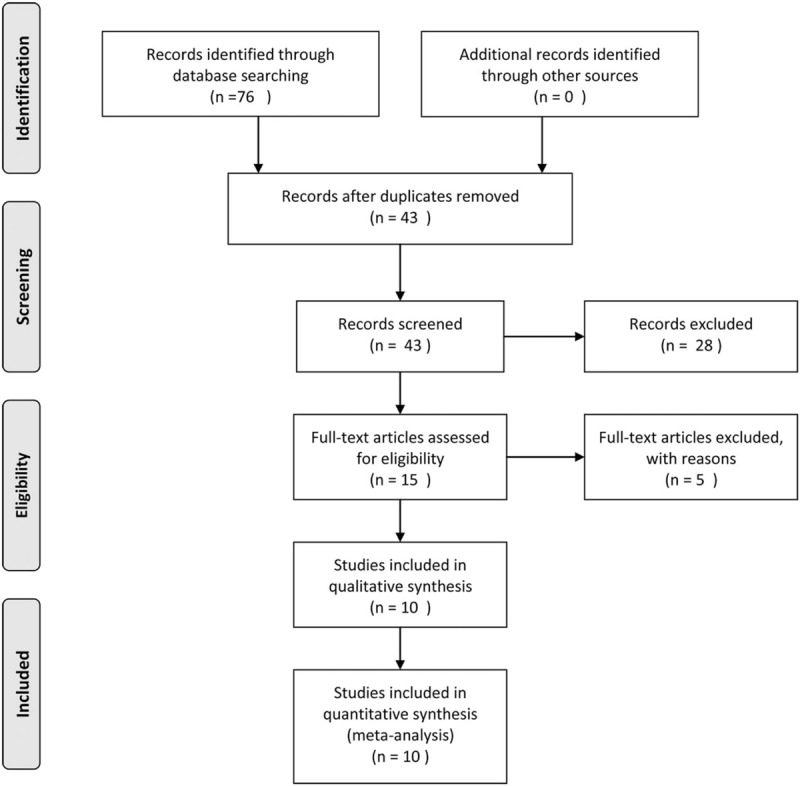
Flow chart of the literature search and selection.

The detailed study selection is shown as Fig. 1. A total of 10 studies with 890 participants were included in our meta-analysis.^[13,14,17,18,23–28]^ The characteristics of the 10 studies were summarized in Table 1. All studies were conducted in China, the study samples ranged from 30 to 168, and the studies were published from 2016 to 2018. The types of carcinoma included non-small cell lung cancer (3 studies), colorectal cancer (2 studies), osteosarcoma (2 studies), gastric cancer (1 study), breast cancer (1 study), and cervical cancer (1 study). All of the NOS scores for eligible studies were ≥6, indicating a high quality for the studies.

**Table 1:**
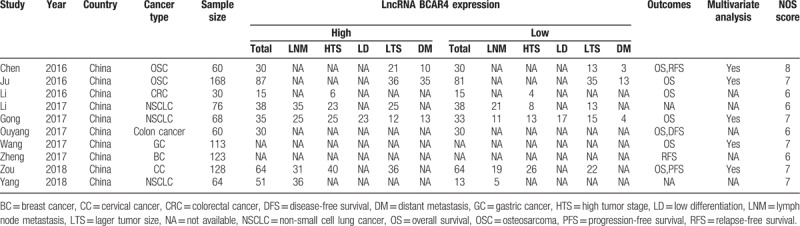
Characteristics of studies included in the meta-analysis.

### 3.2 Association between lncRNA BCAR4 expression and prognosis

**Figure 2:**
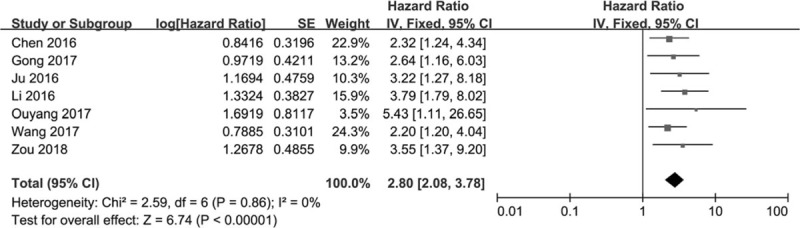
Forest plot of correlation between high lncRNA BCAR4 expression and OS of cancer patients. lncRNA BCAR4 = long non-coding RNA breast cancer anti-estrogen resistance 4; OS = overall survival.

Seven studies were available to assess the effect of lncRNA BCAR4 expression on overall survival (OS). As shown in Fig. 2, statistical analyses revealed that high lncRNA BCAR4 expression was associated with poor OS (HR 2.80, 95% CI: 2.08–3.78; *P* < .001, *I*^2^ = 0%). We then performed a subgroup analysis according to cancer type and analysis type. As shown in Table 2, lncRNA BCAR4 overexpression was correlated with unfavorable OS in patients with osteosarcoma (HR 2.57, 95% CI: 1.53–4.32; *P* < .001, *I*^2^ = 0%), digestive system cancer (HR 2.89, 95% CI: 1.84–4.54; *P* < .001, *I*^2^ = 0%), and other system malignancies (HR 3.00, 95% CI: 1.61–5.60; *P* < .001, *I*^2^ = 0%). The prognostic values of lncRNA BCAR4 in cancer were assessed based on the multivariate analysis in 5 studies. The pooled results revealed that lncRNA BCAR4 overexpression was an independent prognostic factor for OS of cancer patients (HR 2.56, 95% CI: 1.84–3.58; *P* < .001, *I*^2^ = 0%). Relatively fewer studies reported data for relapse-free survival (RFS), disease-free survival (DFS), and progression-free survival (PFS). The prognostic role of lncRNA BCAR4 for RFS was evaluated in 2 studies (Table 1). Patients with high lncRNA BCAR4 expression possessed a significantly shorter RFS than those with low lncRNA BCAR4 expression (HR 1.77, 95% CI: 1.22–2.55; *P* = .002, *I*^2^ = 0%).

**Table 2:**
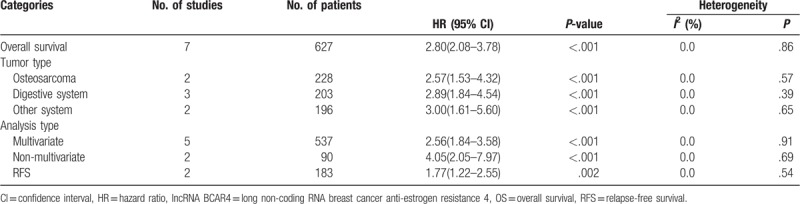
Subgroup analysis of prognostic role of lncRNA BCAR4 on OS and RFS in cancer patients.

### 3.3 Association between lncRNA BCAR4 expression and clinicopathological characteristics

A correlation between lncRNA BCAR4 expression and clinicopathological features were retrieved with OR analysis in 7 studies. The pooled results were shown in Table 3. The pooled results from 5 studies indicated that the high lncRNA BCAR4 expression was not related to tumor size (OR 1.70, 95% CI: 0.88–3.28; *P* < .001, *I*^2^ = 68%). The study by Gong reported that lncRNA BCAR4 expression was not correlated with differentiation (OR 1.80, 95% CI: 0.68–4.79; *P* = .24). However, the pooled results indicated that high lncRNA BCAR4 expression was associated with lymph node metastasis (OR 3.68, 95% CI: 2.25–6.00; *P* < .001, *I*^2^ = 27%), high tumor stage (OR 3.19, 95% CI: 1.98–5.13; *P* < .001, *I*^2^ = 0%), and distant metastasis (OR 3.83, 95% CI: 2.15–6.82; *P* < .001, *I*^2^ = 0%). Therefore, this meta-analysis demonstrated that high lncRNA BCAR4 expression was associated with advanced clinicopathological characteristics.

**Table 3:**
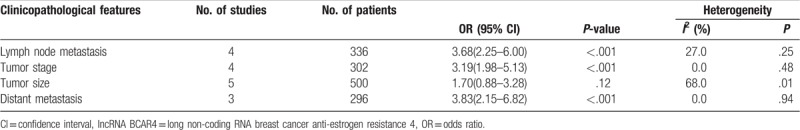
Meta-analysis results for the association between lncRNA BCAR4 expression and clinicopathological features.

### 3.4 Publication bias analysis

**Figure 3:**
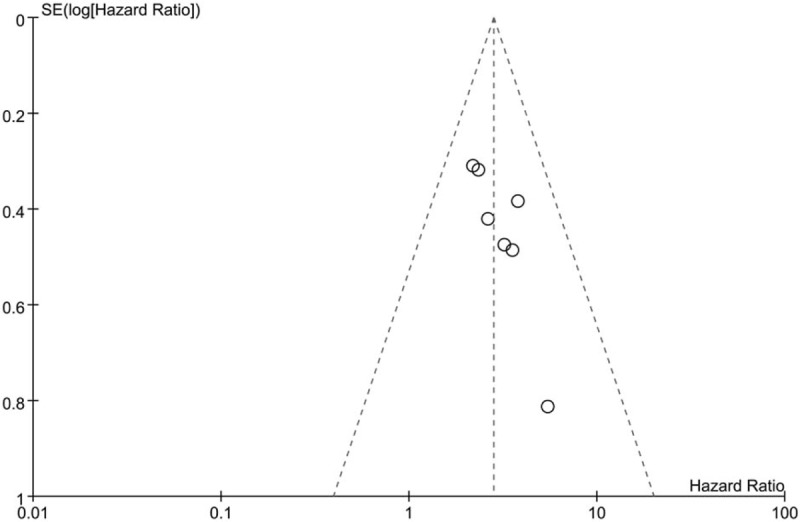
Funnel plot analysis of studies evaluating the relation between lncRNA BCAR4 expression and OS. lncRNA BCAR4 = long non-coding RNA breast cancer anti-estrogen resistance 4; OS = overall survival.

Visual inspection of the funnel plot for the relation of lncRNA BCAR4 expression with OS did not reveal obvious publication bias in our meta-analysis (Fig. 3). Due to the limited number of studies, the publication biases for clinicopathological characteristics were not assessed.

## 4 Discussion

An increasing number of studies indicated that differentially expressed lncRNAs were associated with the development and progression of cancer. LncRNA BCAR4 was first identified as anti-estrogens-resistant in breast cancer cells,^[8]^ and it was located on chromosome 16p13.13. Previous studies have shown that lncRNA BCAR4 was overexpressed in several types of tumors and act as an oncogene in tumors.^[29–32]^ Godinho et al^[11]^ indicated that lncRNA BCAR4 contributed to breast tumor aggression and tamoxifen resistance by regulating the ERBB2/ERBB3 signaling pathways. Chen et al^[13]^ demonstrated that the upregulation of lncRNA BCAR4 expression could promote the proliferation and migration of osteosarcoma cells through activating GLI2-dependent gene transcription. The study by Li et al^[24]^ showed that lncRNA BCAR4 affected the invasion and metastasis of NSCLC cells by acting on epithelial-mesenchymal transition (EMT) pathway. Ouyang et al^[25]^ found that overexpression of lncRNA BCAR4 promoted cell proliferation and migration via activation of Wnt/β-catenin signaling pathway. Wang et al^[26]^ reported that lncRNA BCAR4 could promote drug resistance of gastric cancer by regulating the expression of β-catenin through Wnt signaling pathway. Zheng et al^[27]^ demonstrated that high expression of lncRNA BCAR4 was related to poor survival of breast cancer via Yes-associated protein (YAP)-BCAR4-glycolysis axis.

Recently, it was reported that lncRNA BCAR4 was correlated with clinicopathological features and prognosis of patients with cancer. We conducted a meta-analysis to determine the prognostic value of lncRNA BCAR4 in cancer patients. Ten studies with 890 patients were included in this meta-analysis. The pooled results demonstrated that lncRNA BCAR4 overexpression was significantly related to poor prognosis and could be used as an unfavorable prognostic biomarker in cancer patients. Moreover, the relation between lncRNA BCAR4 and clinicopathological parameters was assessed. The pooled results indicated that overexpression of lncRNA BCAR4 was associated with lymph node metastasis, high tumor stage, and distant metastasis; however, no relation was determined between lncRNA BCAR4 and tumor size.

Several limitations should be taken into account while interpreting the results of this meta-analysis. First, all included studies were from China so that our results may not be globally applicable. Second, no HRs were provided in some studies; therefore, we extracted the data from the Kaplan–Meier survival curves, which could cause errors. Third, the cancer types and sample sizes of included studies were relatively small. Thus, further high-quality studies with large sample sizes are needed to verify the function of lncRNA BCAR4 in various cancer.

## 5 Conclusions

In conclusion, lncRNA BCAR4 overexpression is significantly related to lymph node metastasis, high tumor stage, and distant metastasis; moreover, high lncRNA BCAR4 expression is associated with poor OS in cancer patients. Thus, lncRNA BCAR4 may be a novel prognostic biomarker in cancer patients. However, further high-quality studies are needed to support this study.

## Acknowledgments

The authors thank all subjects and all staff who participated in this study.

## Author contributions

**Data curations:** Yang Meng, Yu-Lan Liu.

**Formal analysis:** Yang Meng.

**Investigation:** Yang Meng.

**Methodology:** Yu-Lan Liu.

**Resources:** Tao Fu.

**Software:** Yang Meng.

**Visualization:** Kai Li.

**Writing – original draft:** Yang Meng, Yu-Lan Liu.

**Writing – review & editing:** Kai Li, Tao Fu.
